# Enantioselective Discrimination of Histidine by Means of an Achiral Cubane-Bridged Bis-Porphyrin

**DOI:** 10.1021/acs.langmuir.1c02377

**Published:** 2021-11-16

**Authors:** Simona Bettini, Nitika Grover, Michela Ottolini, Cornelia Mattern, Ludovico Valli, Mathias O. Senge, Gabriele Giancane

**Affiliations:** † Department of Biological and Environmental Sciences and Technologies, DISTEBA, University of Salento, Via per Arnesano, Lecce 73100, Italy; ‡ Consorzio Interuniversitario Nazionale per la Scienza e, Tecnologia dei Materiali, INSTM, Via G. Giusti, 9, Firenze 50121, Italy; § School of Chemistry, Chair of Organic Chemistry, Trinity Biomedical Sciences Institute, Trinity College Dublin, The University of Dublin, 152−160 Pearse Street, Dublin 2, Ireland; ∥ Department of Engineering of Innovation, Campus University Ecotekne, University of Salento, Via per Monteroni, Lecce 73100, Italy; ⊥ Department of Cultural Heritage, University of Salento, Via D. Birago, Lecce 73100, Italy

## Abstract

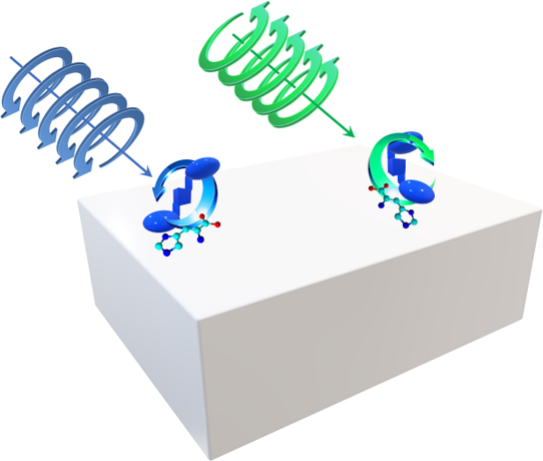

A Langmuir film of cubane-bridged bisporphyrin (**H**_**2**_**por-cubane-H**_**2**_**por**) at the air/water interface was developed and characterized. The floating film was successfully employed for the chiral discrimination between l- and d-histidine. The enantioselective behavior persisted after the deposition of the film on a solid support using the Langmuir–Schaefer method. Distinct absorption and reflection spectra were observed in the presence of l- or d-histidine, revealing that conformational switching was governed by the interaction between **H**_**2**_**por-cubane-H**_**2**_**por** and the histidine enantiomer. The mechanism of chiral selection was investigated using an *ad hoc* modified nulling ellipsometer, indicating the anti-conformation was dominant in the presence of l-histidine, whereas the presence of d-histidine promoted the formation of *tweezer* conformation.

## Introduction

Chiral discrimination is a chemical interaction phenomenon, by which a receptor recognizes a specific enantiomer of substrate molecules.^1^ Different enantiomers of the same molecule may exhibit different properties under physiological conditions. Furthermore, the human olfactory and gustatory systems use chiral interactions. Hence, d-asparagine tastes sweet, and l-asparagine tastes bitter. The toxicity and/or efficacy of a molecule depends upon the ratio of enantiomers or the presence of a specific enantiomer in a sample. The true medical importance of a pure enantiomer was realized through the thalidomide tragedy, a global medical disaster in the late 1950s.^2^ In 1979, Blaschke and co-workers discovered that *R*-thalidomide exhibits a therapeutic effect, whereas *S*-enantiomer is a teratogen.^3^ Hence, the birth defects could have been avoided if only the *R*-enantiomer had been used instead of a racemic mixture. Since then, chemists from all over the world have devoted tremendous efforts to the field of chiral discrimination and enantiomeric excess (*ee*) analysis.^1,4^

In nature, many biomolecules such as amino acids and sugars are present in a chiral form. More than 20 proteinogenic amino acids^5^ are listed and, except for glycine, all of them are chiral molecules. In humans and other vertebrates, proteins are mainly composed of l-amino acids.^6^l-amino acids participate in several biological processes such as impulse neurotransmission^7^ and regulatory functions of cells.^8^ Nature preferentially uses the l-amino acids for the ontogenesis of living organisms. Exceptionally, d-amino acids are present in deep ocean microorganisms^9^ and cell walls of Gram-positive bacteria.^10^ The reason for the asymmetric chiral approach used in many natural processes is fascinating, controversial, and still unknown.

l-histidine contains an α-amino group, (−NH_3_^+^ under physiological conditions), a carboxyl group (−COO^–^ under physiological conditions), and an imidazole side chain. Histidine is the precursor of histamine, a vital inflammatory agent in immune responses.^11^l-histidine plays several biological roles; it is able to bind to iron centers in hemoglobin and myoglobin;^12^ it is often present in the active sites of metalloenzymes (such as carbonic anhydrase and cytochromes)^12^ and acts as an antioxidant^13^ and a mitochondrial glutamine transporter inhibitor.^14^ For a long time, histidine was not considered as an essential amino acid. A lack of histidine in the adult daily diet induces the metabolization of hemoglobin and carnosine,^12^ resulting in low hemoglobin concentration in blood and low carnosine in muscular tissues.^15^ In pharmacological applications, l-histidine is used to prevent fatigue during physical efforts^16^ and to cure aging-related disorders,^17^ dermatitis,^18^ inflammatory, and ocular diseases.^19^d-Histidine plays a marginal role in physiology. It was proposed as a protecting agent against infections from *Bacillus anthracis* spores.^20^ Antifungal activities of d-histidine were also reported in the literature.^21^

**Figure 1:**
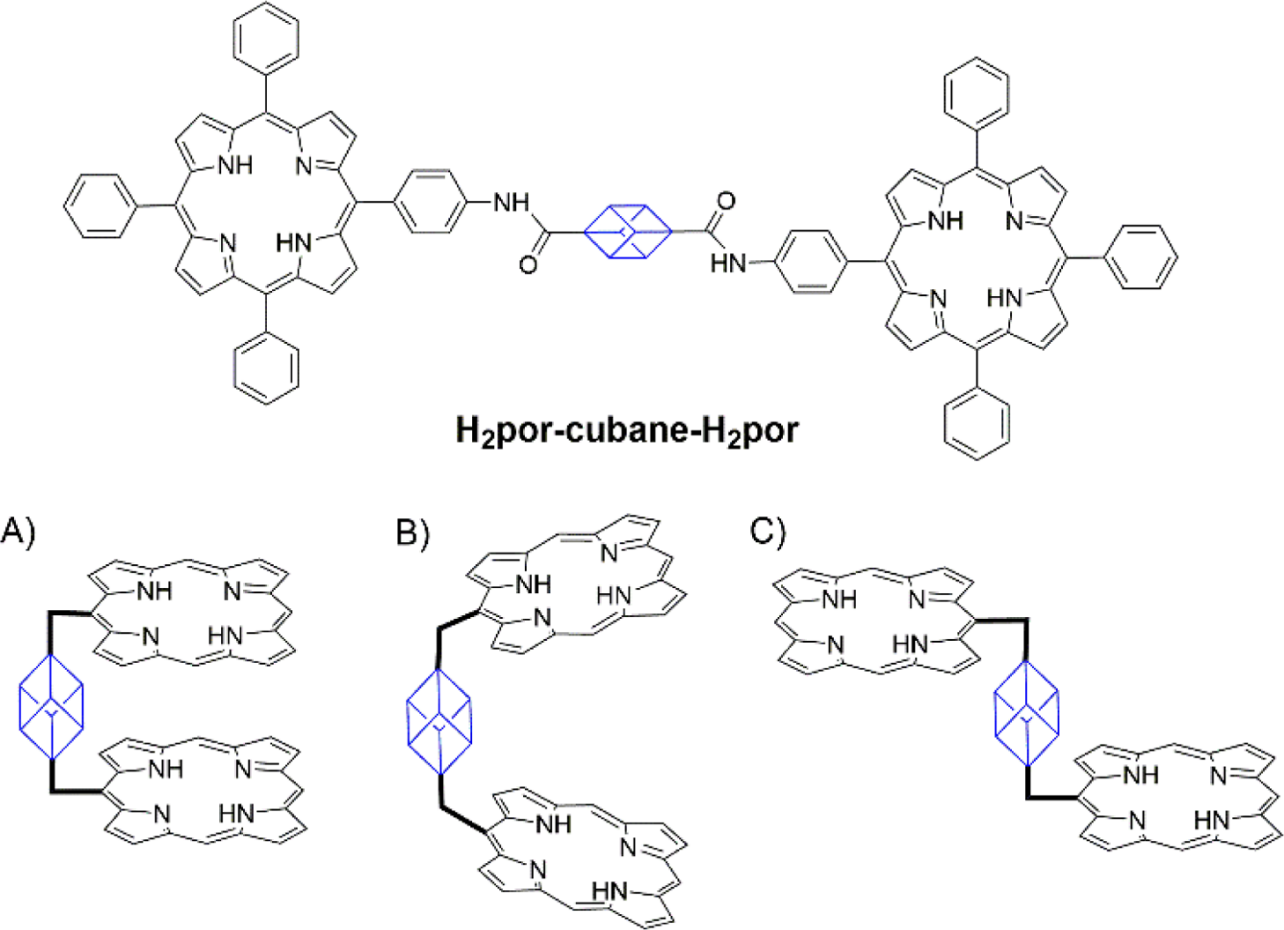
Chemical structure of the cubane-bridged bisporphyrin (**H**_**2**_**por-cubane-H**_**2**_**por**) and schematic representation of the possible configurations adopted by the **H**_**2**_**por-cubane-H**_**2**_**por**. (A) Syn-form, (B) *tweezer* configuration, and (C) anti-form.

Although l- and d-histidine play different roles in nature, the chiral discrimination between their enantiomers is difficult. The lock and key mechanism is the most common approach used for chiral detection.^22^ The three-point interaction model is another method typically used to design active layers for chiral recognition.^23^ Additionally, basket molecules (such as cyclodextrins)^24^ and supramolecular systems are also used as an enantioselective receptor.^25^ Various aromatic systems have been used for the (enantio)differentiation of (di)amines and (amine)alcohols in solution and in the solid state.^26−29^ Porphyrins are the pigments of life, performing a variety of roles such as oxygen transport, electron transfer, oxidation reactions, and photosynthesis in nature.^30^ The porphyrin scaffolds have been used as “building blocks” in molecular engineering of structurally defined multichromophoric arrays. Furthermore, it has been demonstrated that bismetalloporphyrins act as excellent sensors for the detection of a variety of guests, including aromatic amines.^31^ Furthermore, a few reports on chiral detection of amino acids using chiral porphyrins have been published; however, reports on the enantioselective detection using an achiral (free base) porphyrin receptor are scarce. In the present contribution, a method to recognize l-histidine by means of a nonchiral organic molecule is proposed. To this end, we have used an achiral cubane-bridged-bisporphyrin (**H**_**2**_**por-cubane-H**_**2**_**por**, Figure 1)^32^ to form supramolecularly arranged thin films employing the Langmuir–Schaefer (LS) method.^33^ The LS film was used as a receptor for the chiral discrimination between l- and d-histidine. The spectroscopic investigations indicate that each enantiomer is able to stabilize only one kind of bisporphyrin conformer. In particular, l-histidine favors the left-handed configuration of the bisporphyrin derivative.

## Materials and Methods

The **H**_**2**_**por-cubane-H**_**2**_**por** and the **(Zn)por-cubane-H**_**2**_**por** (Figure S1) were synthetized according to the procedure reported in the literature.^32^

A NIMA-KSV trough equipped with a Brewster angle microscope^34^ and a reflection spectrophotometer were used to record the isotherm curve surface pressure *vs* area per molecule of the floating film, and the barrier speed was set at 5 mm min^–1^ for all the Langmuir experiments. The **H**_**2**_**por-cubane-H**_**2**_**por** solution was obtained by dissolving 0.1 mg in 10 mL of chloroform (10^–4^ M), and 150 μL was spread at the air/water subphase interface by means of a glass syringe. Reflection spectra were obtained as a difference between the reflection intensity of the pure subphase and the subphase covered by the floating film that is directly proportional to the absorbance of the floating thin film.^35^

The floating films were transferred to different solid supports (quartz and silicon dioxide) by means of the LS method,^36^ the horizontal variation of the most known Langmuir–Blodgett technique.^37^ For ellipsometer measurements and UV–visible characterization, four LS runs were deposited.

UV–visible spectroscopy was performed with a PerkinElmer 650 spectrophotometer, and an EP4 Accurion-modified nulling ellipsometer was used to monitor the optical activity of the LS films. The angle of the compensator element of the nulling ellipsometer, a λ/4 phase retarder, was set to 0°, and the polarizer was fixed first at 45° to obtain left-handed circularly polarized incident light and at −45° to obtain right-handed circularly polarized incident light. The circularly polarized light was incident directly on the LS films deposited on silicon slides, and a multi-wavelength source was used to investigate the visible range. This configuration was necessary for monitoring the optical activity of the transferred thin films because the optical density related to a LS film obtained by 4 LS runs appears to be too low to be characterized by circular dichroism. Unfortunately, the possibility to deposit a larger number of layers has to be excluded because when the number of LS runs increases, the optical absorption profile of the deposited film strongly changes the relative intensities of the syn-, anti-, and *tweezer* forms (Figure S2). This evidence prompted us to work with a very low number of LS layers in order to minimize the effect of stacking process on the detection mechanism.

## Results and Discussion

**H**_**2**_**por-cubane-H**_**2**_**por** molecules can adopt three different conformations called syn-, anti-, and *tweezer* (Figure 1). The three conformers are characterized by different positions of the maximum absorption of the Soret band that red-shifts from the closed (syn-) to the opened (anti-) form.^38^

**Figure 2:**
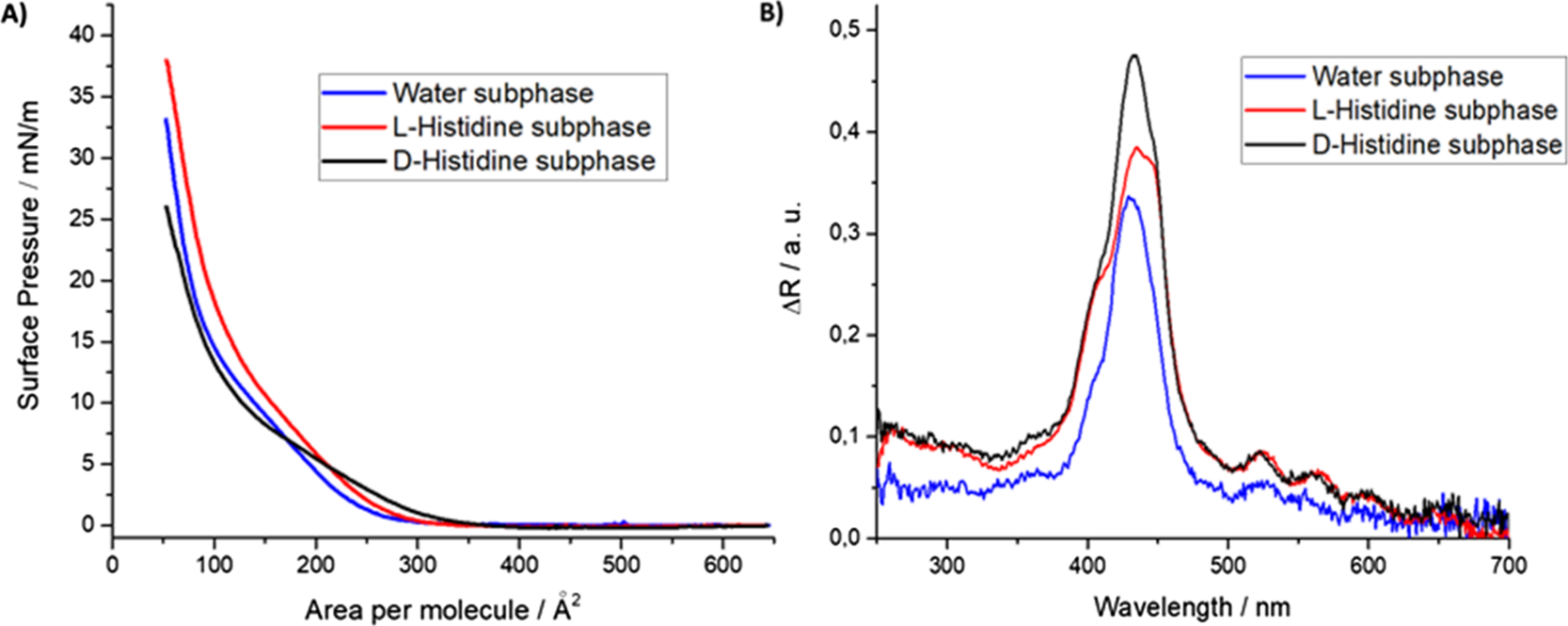
(A) Comparison of the three isotherm curves’ surface pressure vs area per molecule recorded for a **H**_**2**_**por-cubane-H**_**2**_**por** Langmuir floating film on ultrapure water subphase (blue line), aqueous subphase containing l-histidine (10^–4^ M) (red line), and aqueous subphase containing d-histidine (10^–4^ M) (black line). (B) Reflection spectra of **H**_**2**_**por-cubane-H**_**2**_**por** floating film on ultrapure water subphase (blue line), aqueous subphase containing l-histidine (10^–4^ M) (red line), and aqueous subphase containing d-histidine (10^–4^ M) (black line). All the spectra were acquired at a surface pressure of 20 mN m^–1^.

**Figure 3:**
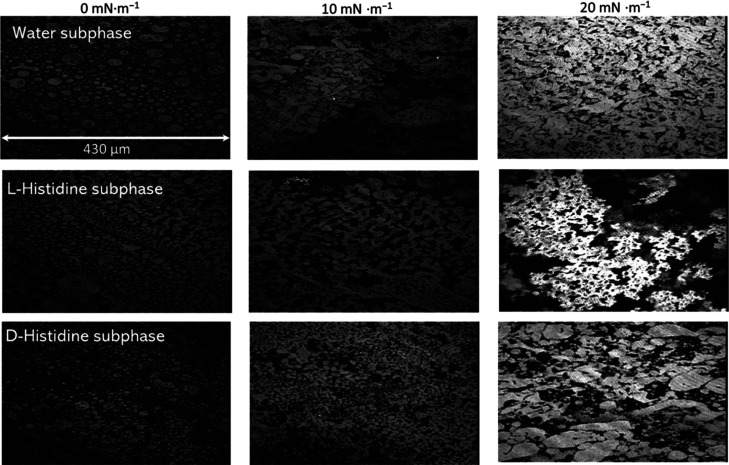
Brewster Angle Microscopy carried out on H_2_por-cubane-H_2_por floating films spread on ultrapure water subphase (top line), on subphase containing l-histidine (10^–4^ M) (middle row) and, in lower line, on subphase containing d-histidine (10^–4^ M).

Chloroform solutions of **H**_**2**_**por-cubane-H**_**2**_**por** were spread at the air/water interface by means of a gas tight syringe (150 μL) and, after the chloroform evaporation, the isotherm curve surface pressure *vs* area per molecule was recorded at a constant barrier speed of 5 mm min^–1^ (black line in Figure 2A). A long pseudo-gaseous phase is recorded, suggesting that the behavior of the molecules of the floating film is far enough from the ideal amphiphilic molecules forming the typical Langmuir floating films.^39^ An abrupt slope change is recorded at approx. 250 Å^2^ molecule^–1^, and a further variation of curve profile is evident at approx. 15 mN m^–1^. Both variations are related to a new rearrangement of the molecules at the air–water interface (blue line, Figure 2A). The value of the limiting area per molecule calculated for the first slope change of the isotherm curve (approx. 250 Å^2^) agrees with the area occupied by a single 5,10,15,20-tetraphenylporphyrin;^40^ hence, it is indicative of **H**_**2**_**por-cubane-H**_**2**_**por** in the syn-form. For further clarification, Brewster angle microscopy (BAM) was performed (Figure 3), and the results suggest that a nonhomogenous covering of the surface is obtained for the **H**_**2**_**por-cubane-H**_**2**_**por** floating film. Therefore, the extrapolated area per molecule value of 250 Å^2^ is not unequivocally connected to the syn- or anti-form of the bisporphyrin. The second branch of the isotherm curve gives a limiting area per molecule of approx. 112 Å^2^; this confirms the formation of a multilayered floating film.^38^ Preliminary evidence of the selective interaction between **H**_**2**_**por-cubane-H**_**2**_**por** molecules and histidine enantiomers was readily evident by the Langmuir curves in red and black in Figure 2A, recorded for l- and d-enantiomers, respectively. The curve trend recorded for the **H**_**2**_**por-cubane-H**_**2**_**por** Langmuir layer spread on l-histidine containing aqueous subphase (10^–4^ M) is very similar to that one recorded in the case of an ultrapure water subphase, even though a higher value of surface pressure is reached (approx. 38 mN m^–1^ for the film floating on l-histidine containing subphase and about 32 mN m^–1^ for **H**_**2**_**por-cubane-H**_**2**_**por** on ultrapure water subphase). In the presence of the d-histidine containing subphase (10^–4^ M), the isotherm Langmuir curve appears drastically different from both the **H**_**2**_**por-cubane-H**_**2**_**por** layer spread on ultrapure water subphase and bisporphyrins Langmuir layer floating on 10^–4^ M l-histidine water solution. BAM images (Figure 3) confirm that floating films obtained on the three different subphases are morphologically different and that a nonuniform covering of the interface is obtained in the three Langmuir experiments.

The chloroform solution of **H**_**2**_**por-cubane-H**_**2**_**por** at the air/water subphase showed a Soret band at 432 nm (Figures 2B and S3). The introduction of l-histidine at low surface pressure to the porphyrin floating layer did not alter the position of the Soret band, indicating that the **H**_**2**_**por-cubane-H**_**2**_**por** molecules exist in the *tweezer* form (Figure S4). With the increasing surface pressure, the Soret band intensity (Δ*R*) increased, and a shoulder appeared at 400 nm. The band at 400 nm corresponds to the closed (syn-) conformer of **H**_**2**_**por-cubane-H**_**2**_**por**. A further increase of the surface pressure by the barrier compression induced an enhancement of the intensity band at 400 nm and a new strong absorption band appeared at 445 nm, corresponding to the anti-conformer (Figure 2B). In the presence of d-histidine (Figure S5), the main absorption band is still located at 432 nm and the intensities of two signals at 400 and 445 nm do not substantially increase under the barrier action; hence, the *tweezer* conformation was retained. Overall, the reflection spectra demonstrated that two histidine enantiomers interact with the **H**_**2**_**por-cubane-H**_**2**_**por** molecules at the air/subphase interface and the interaction mechanisms are governed by the chiral form of the amino acid.

**Figure 4:**
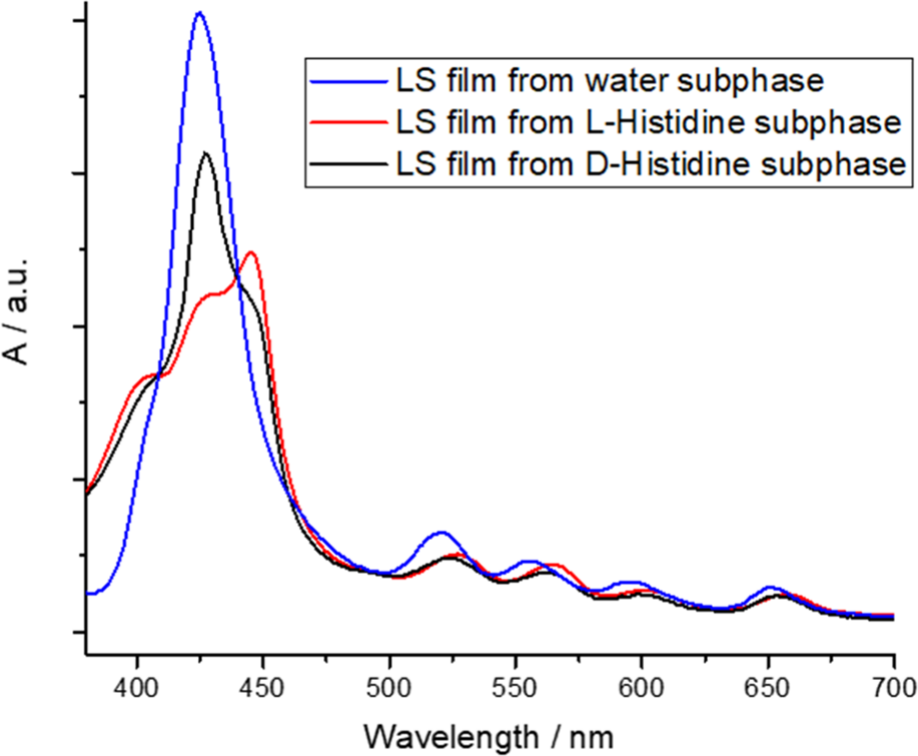
Visible spectra of LS films (4 runs) of **H**_**2**_**por-cubane-H**_**2**_**por** transferred from ultrapure water (blue line), aqueous subphase containing l-histidine (10^–4^ M) (red line), and aqueous subphase containing d-histidine (10^–4^ M) (black line).

The floating films from water, l-histidine, and d-histidine subphases were transferred on a solid substrate using the LS method. The obtained thin solid films were characterized by means of UV–visible spectroscopy (Figure 4). The differences among the three LS layers are evident: the LS film deposited from the ultrapure water subphase showed a well-defined band at 425 nm; the thin film transferred using d-histidine containing subphase was characterized by a pronounced shoulder at 445 nm and a less intense one at 402 nm. In the case of the thin film obtained from the l-histidine subphase, an absorption band at 445 nm is dominated in the whole absorption spectrum, even though the bands at 425 and 400 nm are evident. Furthermore, the Q-bands at 521, 556, 594, and 650 nm are red-shifted for **H**_**2**_**por-cubane-H**_**2**_**por** LS films obtained from both l- and of d-histidine-containing subphase.

**Figure 5:**
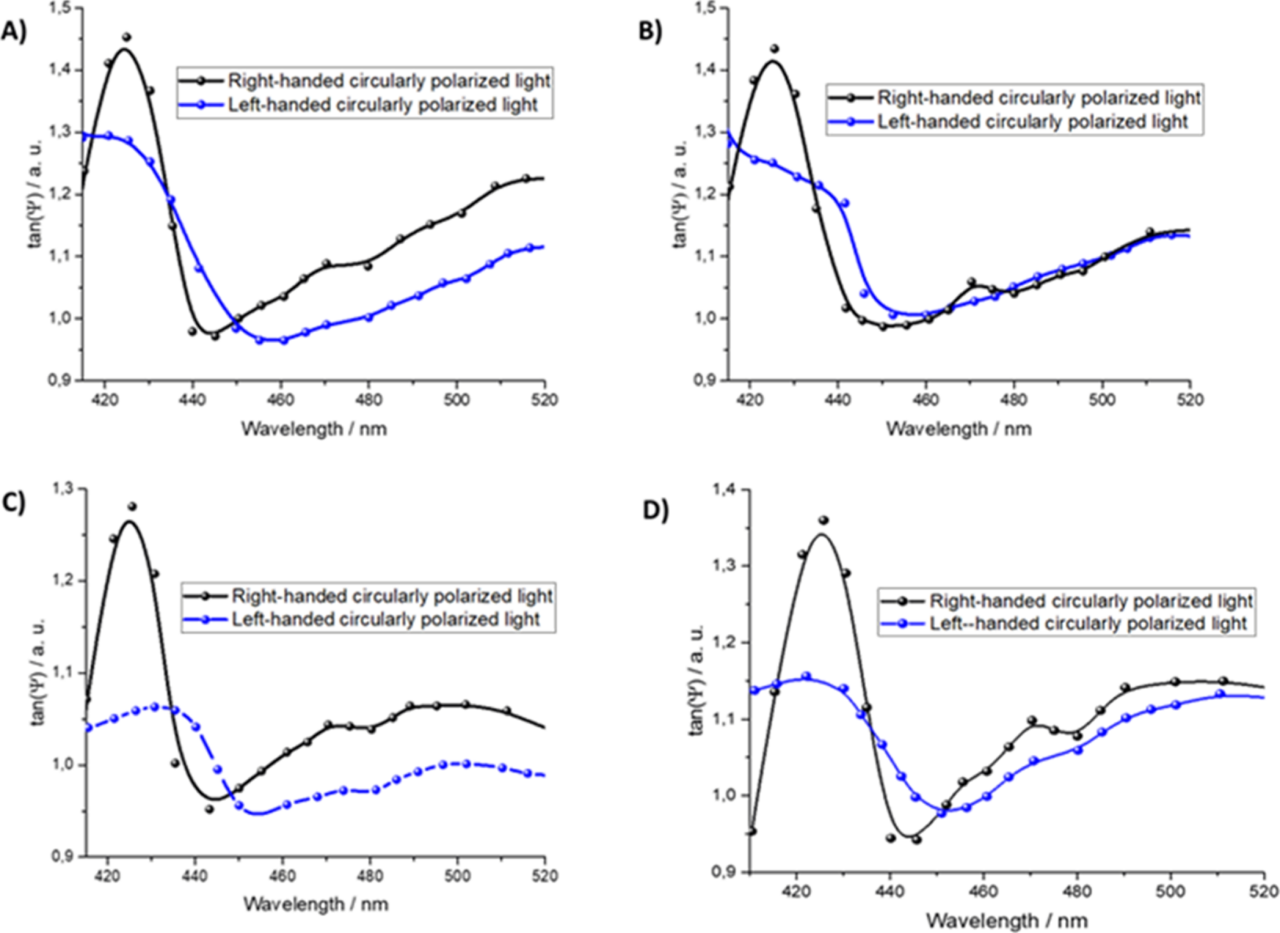
Ellipsometer measurements performed using left-handed circularly polarized light (in blue in all the images) and right-handed circularly polarized light (black lines). Four samples were investigated: (A) 4 runs of LS film transferred from ultrapure water subphase; (B) 4 runs of LS film transferred from water subphase containing l-histidine (10^–4^ M); (C) 4 runs of LS film transferred from water subphase containing d-histidine (10^–4^ M); and (D) 4 runs of LS film transferred from water subphase containing racemic solution of D and l-histidine (10^–4^ M).

The optical activity of the **H**_**2**_**por-cubane-H**_**2**_**por** LS films was investigated by means of an ellipsometer set to have left and right circularly polarized incident light.^41^ In an EP4 nulling ellipsometer, the polarizer was set at 45° and the compensator at 0° to obtain a left-handed circularly polarized light; when the polarizer is fixed at −45° and the compensator at 0°, a right-handed circularly polarized is incident on the sample. This approach was used because it allows for the characterization of very thin solid films without any sample treatment. The results obtained using this approach are reported in Figure 5 for the case of LS films deposited from the water subphase (Figure 5A), from l-histidine subphase (Figure 5B), from d-histidine (Figure 5C), and from a histidine racemic solution (Figure 5D).

Ellipsometer measurements suggest that the Langmuir film transferred onto the silicon substrate from the air/ultrapure water subphase is formed both by right-handed and by left-handed conformers. When right-handed light is used, a pronounced band at 425 nm appears, indicating the presence of the *tweezer* form, whereas the presence of left-handed light induces a shoulder at 440 nm, suggesting the presence of the anti-form of **H**_**2**_**por-cubane-H**_**2**_**por** (Figure 5A). These results further confirm the observation obtained from the visible spectra (Figure 4, line blue) and suggest that the *tweezer* and anti-forms are preferentially characterized by clockwise and anticlockwise chirality, respectively. Therefore, the tweezer arrangement is the predominant molecular structure at the air/ultrapure water subphase. The presence of l-histidine in the subphase during the transfer process considerably influences the aggregation state of the thin film’s molecules, as observed in Figure 4 and in Figure 5B. The features observed for the right-handed circularly polarized light show an intense and symmetric band at 425 nm; on the contrary, the left-handed circularly polarized light clearly evidences the band at 440 nm. This result suggests that the presence of l-histidine in the subphase influences the **H**_**2**_**por-cubane-H**_**2**_**por** Langmuir film deposition process. In particular, the formation of a chiral supramolecular adduct (porphyrin/l-amino acid) is favorable. This adduct is preserved during the deposition process and shows a preferential anti-clockwise chirality.

**Figure 6:**
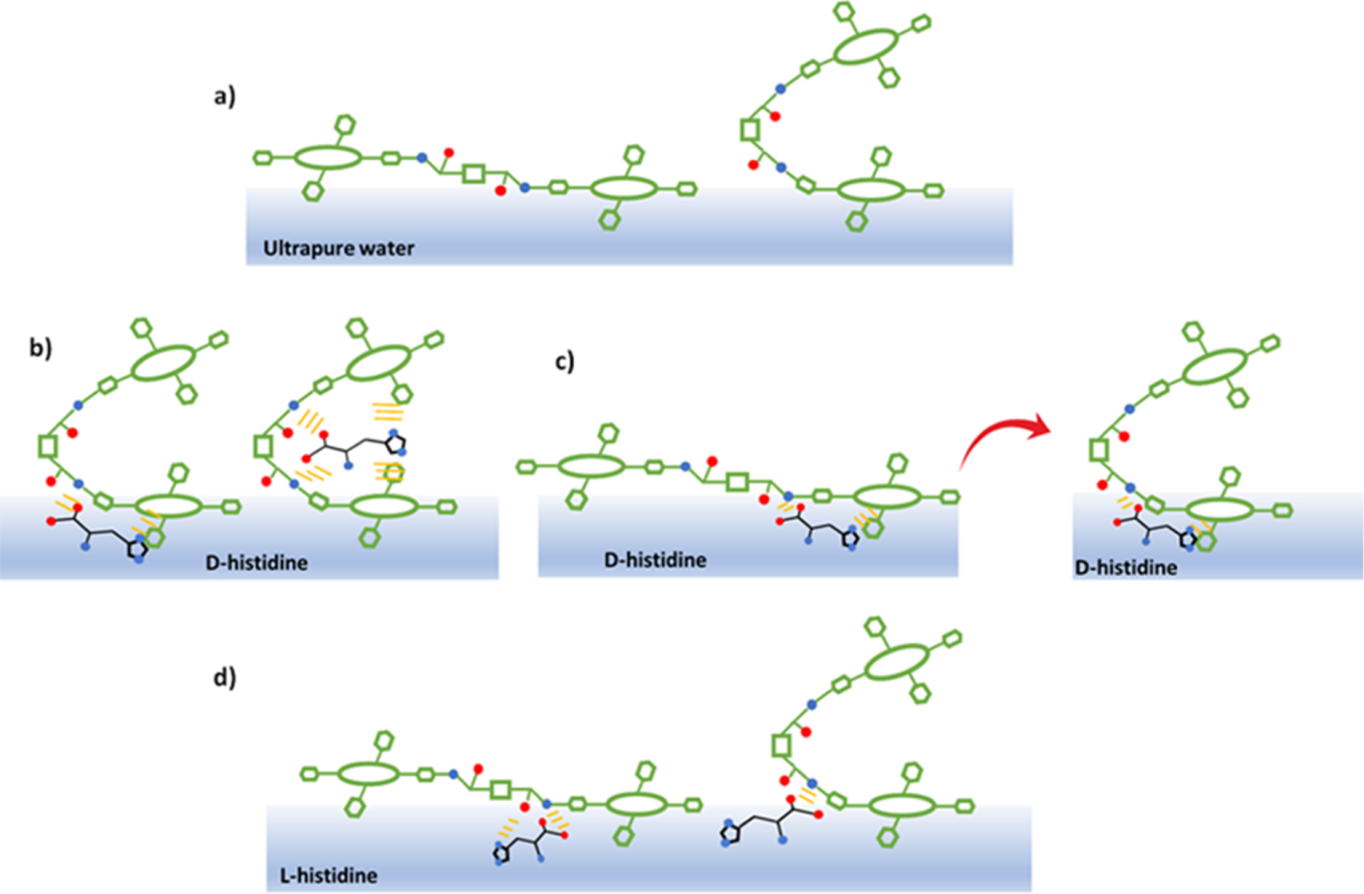
Schematic representation of the plausible interaction mechanisms that govern the configuration changes of **H**_**2**_**Por-cubane-H**_**2**_**Por** at the air/subphase interface (panel a) in the presence of d-histidine (panel b,c) and l-histidine (panel d). The behavior of each bisporphyrin conformer was considered depending on the analyte dissolved in the subphase.

The opposite effect on the porphyrin aggregation is induced by the presence of d-histidine in the subphase (Figure 5C). The number of molecules that interact with the circularly polarized light in a left-handed way (anti-form) decreases, and the number of molecules in *tweezer* conformation (characterized by a clockwise chirality) increases. From the reflection spectra, it can be proposed that at the air/water interface, the porphyrin molecules are arranged in both the *tweezer* and anti-forms (Figure 6, image a). However, the *tweezer* form was found to be predominant at the air/water interface. Furthermore, the deposition procedure preferentially promotes the molecules to change to the *tweezer* form that shows a predominant clockwise chirality.

When the interaction takes place between d-histidine and the *tweezer* porphyrin molecules, no relevant effect on the conformational arrangement can be observed. Furthermore, d-histidine can be accommodated in the bite of the *tweezer* conformer, promoting a stable binding among the amino acids’ carboxylic group and the amide groups on the two sides of the cubane.^31^ The imidazole group of histidine can be trapped between the two macrocycles of the bisporphyrin in *tweezer* form (Figure 6b). The tweezer form, which presents clockwise chirality, is so stabilized by interaction with d-histidine molecules. On the other hand, when d-histidine interacts with the anti-form of **H**_**2**_**por-cubane-H**_**2**_**por** (anti clockwise chiral behavior), the COO^–^ moiety of histidine can interact with the −NH group of the amide bond, while the histidine NH_3_^+^ interacts with the C=O of the amide group (Figure 6c). It is reasonable to propose that the imidazole ring of the dissolved amino acid overlaps with the π-cloud of the porphyrin macrocycle on the subphase interface. As reported for similar systems, a conformational change from the opened to the *tweezer* form of bisporphyrin derivatives can be promoted.^31,42^

When l-histidine is dissolved in the subphase, it can interact with both the anti- and *tweezer* forms (Figure 6d). The anti-form is stabilized by the simultaneous interaction with the imidazole and the carboxylic moieties of l-histidine, ensuring the presence of both the opened and the *tweezer* conformer during the deposition process. This mechanism preserves, contrary to the case of the D enantiomer of the amino acid, the presence of anti-clockwise conformers within the film. In the presence of a racemic histidine solution, ellipsometry suggests that the **H**_**2**_**por-cubane-H**_**2**_**por** molecules in Langmuir film are again arranged both as *tweezer* and anti-conformers with a preference for the former. This behavior can be explained considering that (i) the molecules in *tweezer* and anti-form interacting with d-histidine are stabilized in the tweezer form; (ii) the noninteracting bisporphyrin molecules are preferentially transferred in the tweezer form; (iii) the *tweezer* molecules interacting with l-histidine are stabilized again in the *tweezer* form; and (iv) the molecules arranged as anti-conformer that interact with l-histidine are immobilized on the solid support in the opened form (left-handed). It is worth noting that when the LS film is deposited on the solid substrate from air/ultrapure water subphase, the conformational changes induced by L and d-histidine fluxes are strongly attenuated (Figure S6) due to the larger energy needed to induce a conformational change in the immobilized molecules.

To corroborate the proposed rationale, **H**_**2**_**por-cubane-H**_**2**_**por** floating films were transferred from subphases containing different analytes. In particular, chiral aliphatic amino acids (l- and d-lysine) were used to investigate the role of the aromatic moiety in adduct formation. Additionally, the achiral and smallest amino acid glycine was used to investigate the effect of chirality and steric bulk on the adduct formation. When l- and d-lysine were dissolved in the subphase, no relevant changes were observed in the absorption spectra of LS films. This is the consequence of the absence of imidazole moiety in the lysine (Figure S7A). Similarly, glycine did not induce any changes in the absorption spectrum (Figure S7B). In contrast, the presence of histamine in the subphase strongly affected the absorption profile of the bisporphyrin LS film (Figure S7C). In this case, the opened bisporphyrin form is preserved during the deposition process, as for l-histidine, even though changes are smaller due to the absence of the amide moiety and the geometric arrangement of the analyte. This evidence confirms the crucial role of both the imidazole ring and −NH_2_ group in chiral detection. An aromatic amino acid without the imidazole moiety, phenylalanine, was used to clarify the role of imidazole. The effect of d-phenylalanine on the **H**_**2**_**por-cubane-H**_**2**_**por** molecules of the LS film is very similar to that one observed for d-histidine: the bisporphyrins are pushed to preferentially arrange in *tweezer* form. In contrast to the observations in the case of l-histidine, the effect of l-phenylalanine on the **H**_**2**_**por-cubane-H**_**2**_**por** conformational arrangement is almost negligible (Figure S7D). In this case, the Soret band is sharp and centered at 425 nm, indicating that only one form is preserved (the tweezer form). d-phenylalanine and l-phenylalanine have the same behavior. No chiral discrimination takes place.

According to the mechanism proposed in Figure 6, the imidazole group plays a fundamental role in the interaction with the imide group of the cubane bridge, inducing the stabilization of the opened form of the bisporphyrin molecules.

When the monometallated **(Zn)por-cubane-H**_**2**_**por** was used to detect histidine in the subphase, no conformational changes were observed in the presence of d- and l-enantiomers (Figure S8). The high affinity of the imidazole group toward the Zn(II) center promotes the mixing of porphyrin and histidine subphases.^43^ This preferential mechanism inhibits the supramolecular adduct formation and also supports the interaction pathways described earlier (Figure S9).

## Conclusions

The chiral discrimination of d- and l-histidine was achieved by tuning the conformational switching of a cubane-bridged bisporphyrin (**H**_**2**_**por-cubane-H**_**2**_**por**). Spectroscopic investigations of Langmuir films allowed us to monitor the conformations adopted by the bisporphyrin molecules spread at the air/ultrapure water interface of a Langmuir trough. It was observed that the presence of l- and d-histidine in the subphase (10^-4^ M) greatly influences the floating film behavior. Reflection spectra of floating films in the presence of l-histidine showed the presence of an *anti*-conformation, whereas air/water and d-histidine subphase promoted the *tweezer* conformation. Chiral discrimination was preserved during the deposition process using the LS method. Ultrapure water was used as a subphase for the **H**_**2**_**por-cubane-H**_**2**_**por LS film**. The absorption spectrum of the film exhibited an absorption band at 425 nm, revealing the presence of the *tweezer* conformation. A similar absorption spectrum was obtained when the d-histidine subphase was introduced. In contrast, l-histidine promoted the formation of a predominant band at 440 nm, a characteristic of the *anti*-form of **H**_**2**_**por-cubane-H**_**2**_**por**. A rationale for the chiral selectivity and induced conformational changes was further confirmed by investigating the thin LS films under circularly polarized light. The presence of d-histidine increases the number of molecules that interact with the right-handed circularly polarized light; hence, fewer molecules interact with the left-handed polarized light. The very low number of LS layers did not allow for the use of circular dichroism as a characterization technique and an *ad hoc* modified ellipsometer was used. Furthermore, l-histidine promotes the stabilization of *anti*-conformer of the porphyrin molecules, which preferentially interact with left-handed polarized light. A variety of amino acids including glycine, lysine, histamine, and phenylalanine were used to investigate the effect of different functional groups involved in the interactions with **H**_**2**_**por-cubane-H**_**2**_**por**. We observed that a combined presence of the imidazole ring and NH_2_ groups plays a crucial in chiral discrimination and conformational switching. Overall, this work demonstrated that **H**_**2**_**por-cubane-H**_**2**_**por** can be used to determine the absolute configuration of l- and d-histidine.

## Supporting Information Available

Chemical structure of **H**_**2**_**por-cubane-H**_**2**_**por** and **(Zn)por-cubane-H**_**2**_**por**, influence of the number of LS runs on the spectral profile of **H**_**2**_**por-cubane-H**_**2**_**por**, reflection spectra of the floating film of **H**_**2**_**por-cubane-H**_**2**_**por** spread at air/ultrapure water subphase acquired at different surface pressures, reflection spectra of the floating film of **H**_**2**_**por-cubane-H**_**2**_**por** spread at air/l-histidine aqueous subphase acquired at different surface pressures, reflection spectra of the floating film of **H**_**2**_**por-cubane-H**_**2**_**por** spread at air/d-histidine aqueous subphase acquired at different surface pressures, spectral variation induced by fluxing l- and d-histidine on 4 runs LS film of **H**_**2**_**por-cubane-H**_**2**_**por** transferred from ultrapure water subphase, effect on the **H**_**2**_**por-cubane-H**_**2**_**por** LS film absorption spectrum induced by different analytes dissolved in the subphase: (A) l and d-lysine, (B) glycine, (C) histamine, (D) l and d-phenylalanine, LS films of **(Zn)por-cubane-H**_**2**_**por** transferred from ultrapure water (blue line), from subphases containing l-histidine (10^–4^ M) and d-histidine (10^–4^ M), and schematic representation of the interaction among l and d-histidine with the floating molecules of **(Zn)por-cubane-H**_**2**_**por** (PDF)
