# Supplementary material for: Enantioselective Discrimination of Histidine by Means of an Achiral Cubane-Bridged Bis-Porphyrin
Source: Langmuir. 2021 Nov 16;37(47):13882–9. doi: 10.1021/acs.langmuir.1c02377 (PMC8638291; doi:10.1021/acs.langmuir.1c02377)
Supplement: Supplementary file 1 — la1c02377_si_001.pdf [file la1c02377_si_001.pdf]

SUPPORTING INFORMATION

Enantioselective discrimination of histidine by means of an achiral cubane-bridged bis-porphyrin

Simona Bettini,[ab] Nitika Grover,[c] Michela Ottolini,[d] Cornelia Mattern,[c] Ludovico Valli,[ab] Mathias O. Senge,[c] Gabriele Giancane [be*]*

[a] Department of Biological and Environmental Sciences and Technologies, DISTEBA, University of Salento, Via per Arnesano, 73100 Lecce, Italy

[b] Consorzio Interuniversitario Nazionale per la Scienza e, Tecnologia dei Materiali, INSTM, Via G. Giusti, 9, 50121 Firenze, Italy

[c] School of Chemistry, Chair of Organic Chemistry, Trinity Biomedical Sciences Institute, 152-160 Pearse Street, Trinity College Dublin, The University of Dublin, Dublin 2, Ireland

[d] Department of Engineering of Innovation, Campus University Ecotekne, University of Salento, Via per Monteroni, 73100 Lecce, Italy

[e] Department of Cultural Heritage, University of Salento, Via D. Birago, 73100, Lecce, Italy.

Corresponding Author

* Gabriele Giancane; gabriele.giancane@unisalento.it

* Mathias Senge; mathias.senge@tcd.ie

Table of contents. Figure S1. Chemical structure of **H_2por-cubane-H_2por** (a) and **(Zn)por-cubane-H_2por** (b). Figure S2. Influence of the number of LS runs on the spectral profile of the **H_2por-cubane-H_2por**. Figure S3. Reflection spectra of the floating film of **H_2por-cubane-H_2por** spread at air/ultrapure water subphase acquired at different surface pressures (black line corresponds to 0 mN/m, red line to 5 mN/m and blue line to 20 mN/m). Figure S4. Reflection spectra of the floating film of **H_2por-cubane-H_2por** spread at air/L-histidine aqueous subphase acquired at different surface pressures (black line corresponds to 0 mN/m, red line to 5 mN/m and blue line to 19 mN/m). Figure S5. Reflection spectra of the floating film of **H_2por-cubane-H_2por** spread at air/D-histidine aqueous subphase acquired at different surface pressures (black line corresponds to 0 mN/m, red line to 5 mN/m and blue line to 20 mN/m). Figure S6. Spectral variation induced by fluxing L- and D-histidine on 4 runs LS film of **H_2por-cubane-H_2por** transferred from ultrapure water subphase. Figure S7. Effect on the **H_2por-cubane-H_2por** LS film absorption spectrum induced by different analytes dissolved in the subphase: A) L and D-lysine, B) glycine, C) histamine, D) L and D-phenylalanine. Figure S8. LS films of **(Zn)por-cubane-H_2por** transferred from ultrapure water (blue line), from subphases containing L-histidine (10^{-4} M) and D-histidine (10^{-4} M), red and balck line respectively. Figure S9. Schematic representation of the interaction among L and D histidine with the floating molecules of **(Zn)por-cubane-H_2por**.

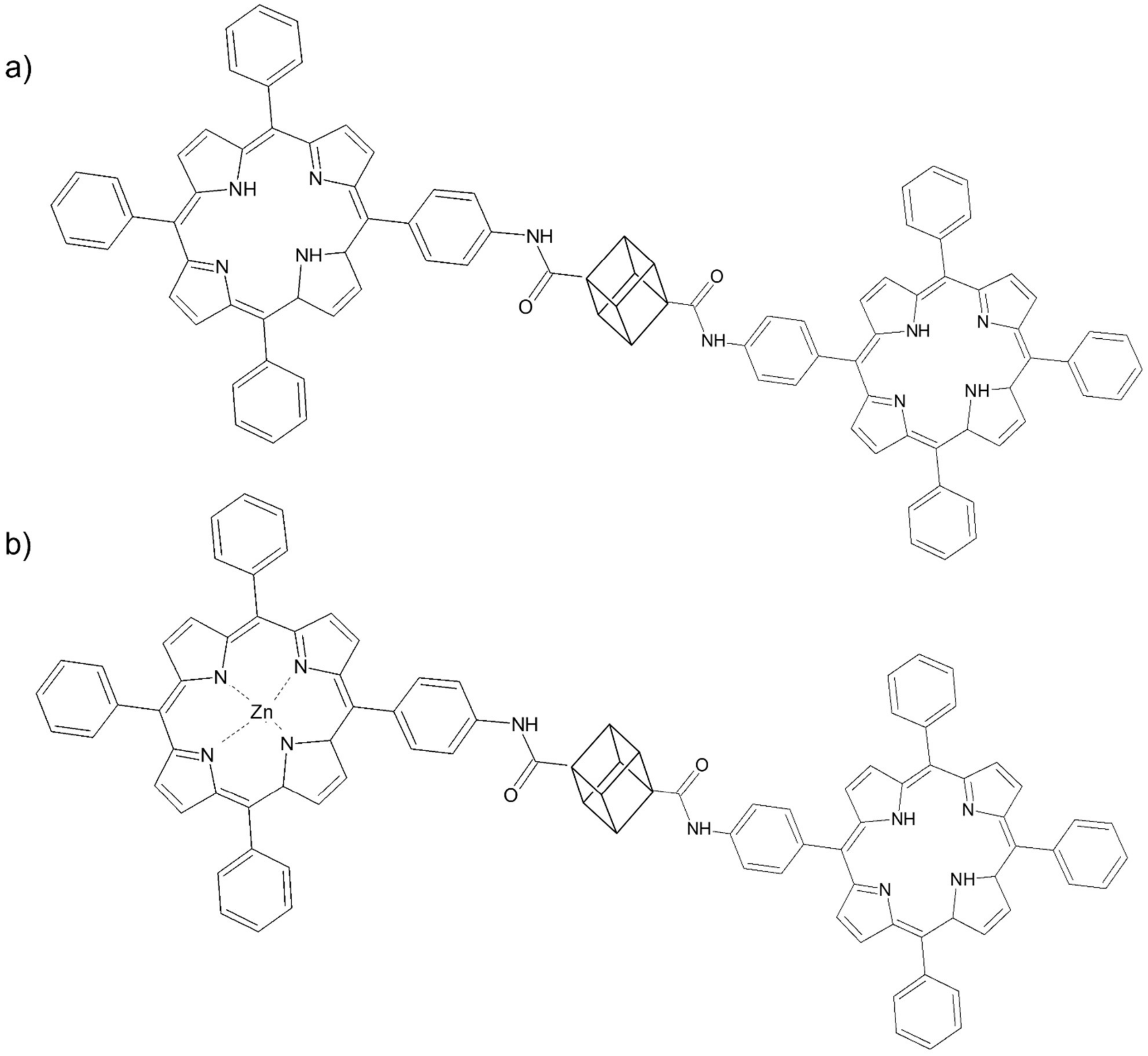

Figure S1. Chemical structure of **H_2por-cubane-H_2por** (a) and **(Zn)por-cubane-H_2por** (b).

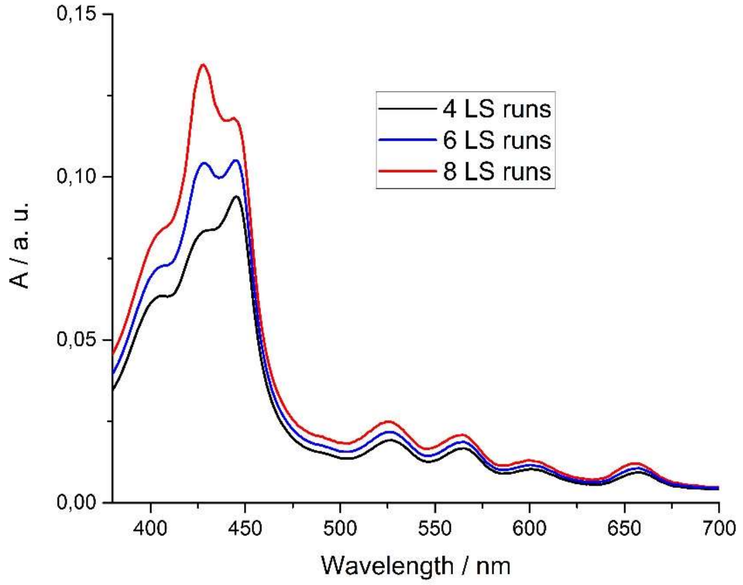

Figure S2. Influence of the number of LS runs on the spectral profile of the **H_2por-cubane-H_2por**.

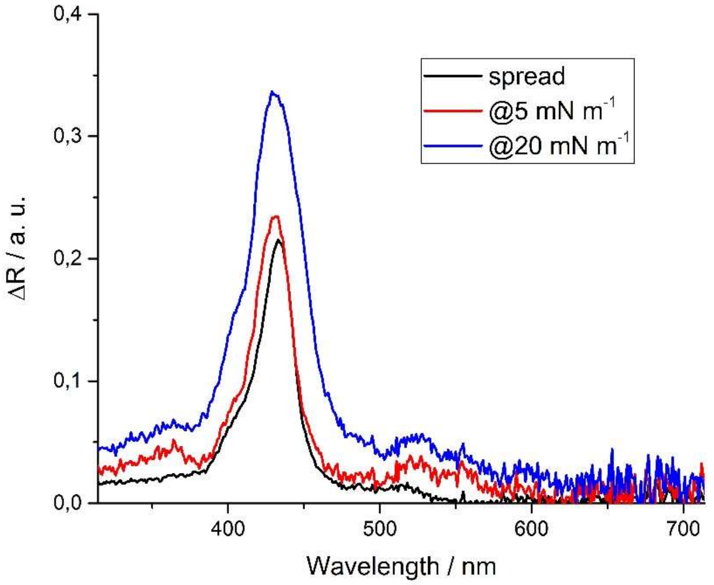

Figure S3. Reflection spectra of the floating film of **H_2por-cubane-H_2por** spread at air/ultrapure water subphase acquired at different surface pressures (black line corresponds to 0 mN/m, red line to 5 mN/m and blue line to 20 mN/m).

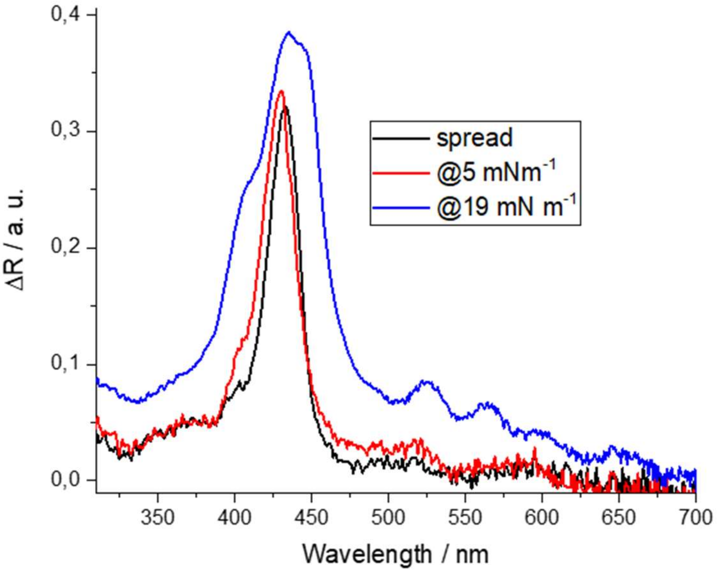

Figure S4. Reflection spectra of the floating film of **H_2por-cubane-H_2por** spread at air/L-histidine aqueous subphase acquired at different surface pressures (black line corresponds to 0 mN/m, red line to 5 mN/m and blue line to 19 mN/m).

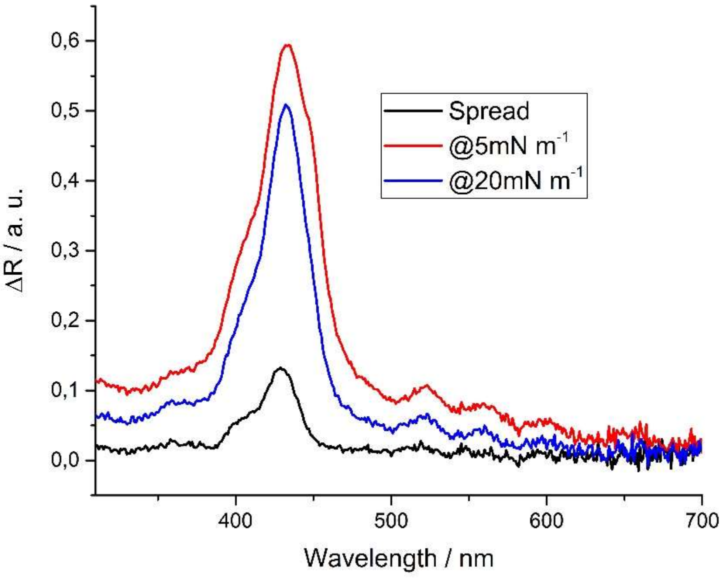

Figure S5. Reflection spectra of the floating film of **H_2por-cubane-H_2por** spread at air/D-histidine aqueous subphase acquired at different surface pressures (black line corresponds to 0 mN/m, red line to 5 mN/m and blue line to 20 mN/m).

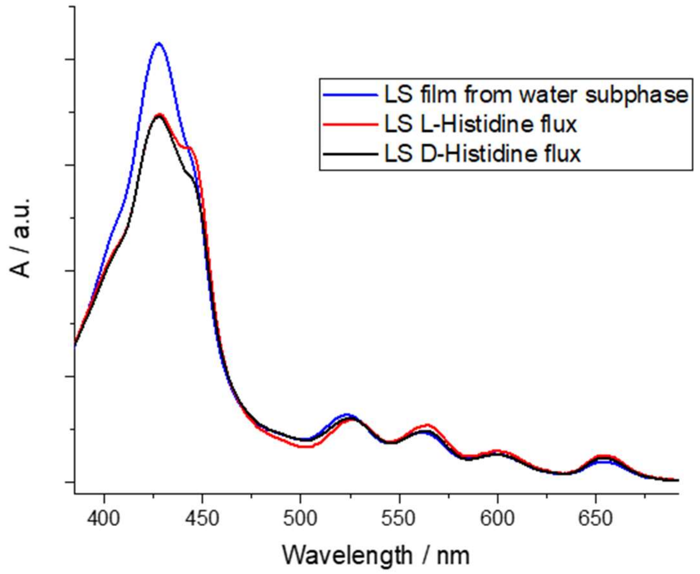

Figure S6. Spectral variation induced by fluxing L- and D-histidine on 4 runs LS film of **H_2por-cubane-H_2por** transferred from ultrapure water subphase.

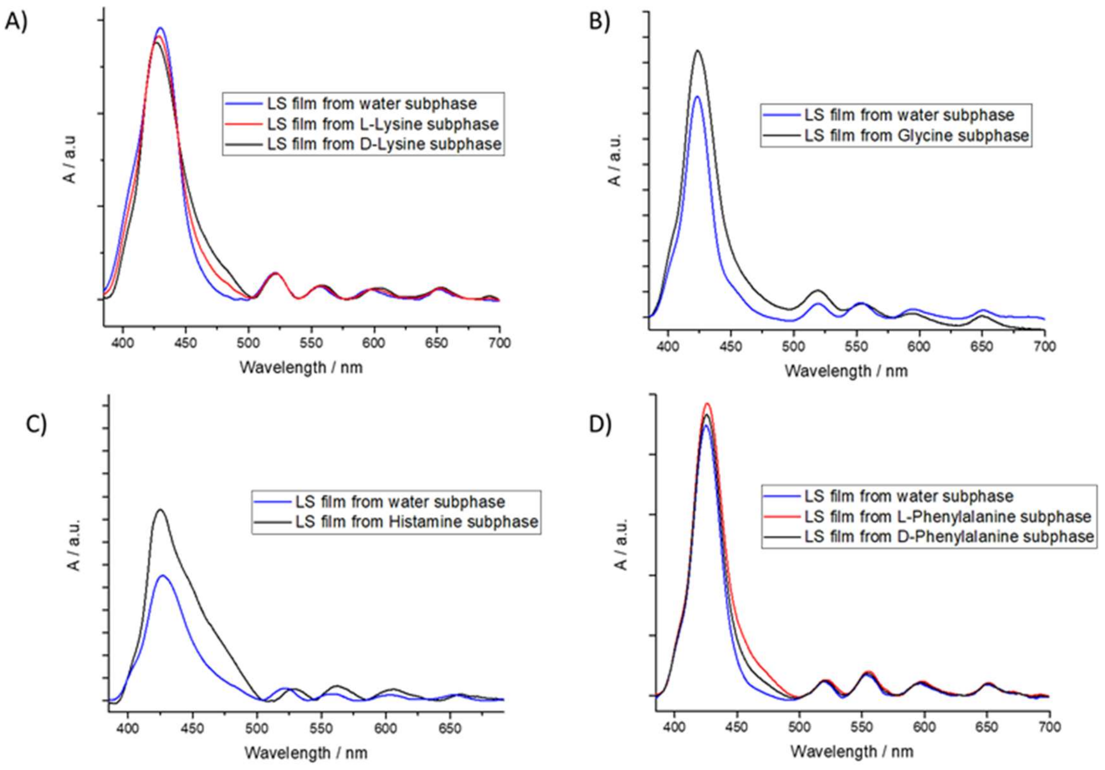

Figure S7. Effect on the **H_2por-cubane-H_2por** LS film absorption spectrum induced by different analytes dissolved in the subphase: A) L and D-lysine, B) glycine, C) histamine, D) L and D-phenylalanine.

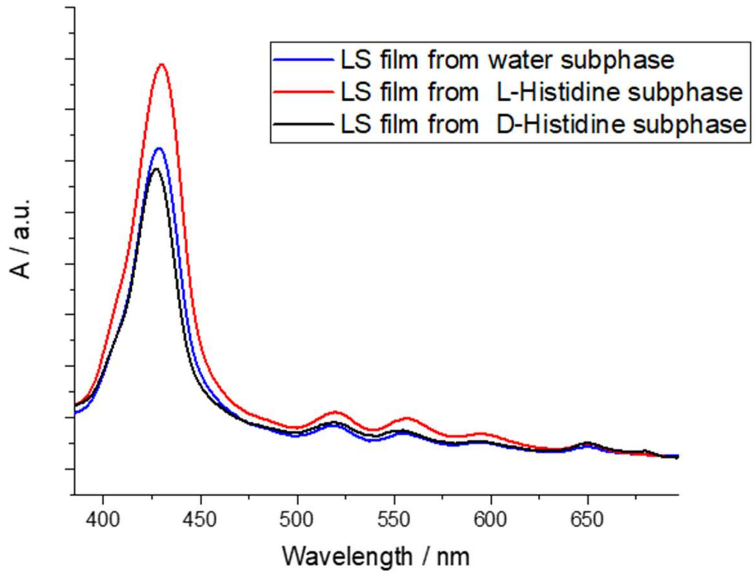

Figure S8. LS films of **(Zn)por-cubane-H_2por** transferred from ultrapure water (blue line), from subphases containing L-histidine (10^{-4} M) and D-histidine (10^{-4} M), red and balck line respectively.

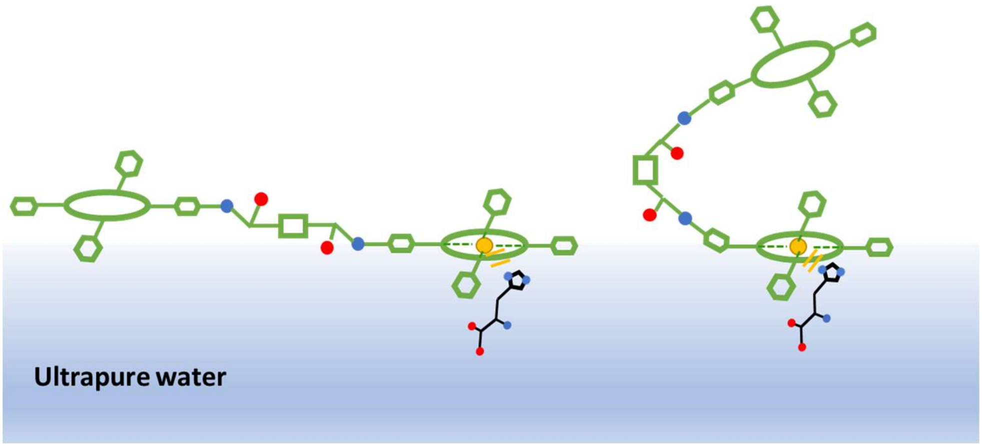

Figure S9. Schematic representation of the interaction among L and D histidine with the floating molecules of **(Zn)por-cubane-H_2por**.
